# The psychological dynamics of the combat sports experience: how the phenomenological specificity of corporal fighting prevents violence and promotes the development of the practitioner

**DOI:** 10.3389/fpsyg.2025.1631471

**Published:** 2025-07-30

**Authors:** Cristiano Roque Antunes Barreira, Thabata Castelo Branco Telles, Carlos Gutiérrez-García, Bernard Andrieu

**Affiliations:** ^1^School of Physical Education and Sport of Ribeirão Preto (EEFERP), University of São Paulo, Ribeirão Preto, Brazil; ^2^University of Maia (UMAIA) and Politechnic Institute of Maia (IPMAIA), N2i, Braga, Portugal; ^3^Department of Physical and Sport Education, Universidad de León, León, Spain; ^4^Paris Institute of Sports and Health Sciences (I3SP), University of Paris, Paris, France

**Keywords:** combat sports, martial arts, corporal fighting, phenomenology, violence, ethical development, lived experience, psychological dynamics

## Abstract

Psychological research on martial arts and combat sports (MA&CS) often neglects the essential specificity of the lived experience of combat, resulting in a lack of a unified conceptual framework. This article proposes a phenomenological perspective to clarify the unique psychological dynamics and developmental potential inherent in corporal fighting. Applying classical phenomenology, and drawing upon empirical-phenomenological research based on interviews across nine MA&CS modalities, we analyze the constitutive structures of this lived experience. We identify corporal fighting as a reciprocal, embodied struggle and foundational, distinct from brawl or play-fighting. Five essential forms (corporal fighting, duel, self-defense, instrumental offensive combat, play-fighting) are distinguished by intentional structures. Traditional, modern, and military martial arts simulate duel, self-defense, and instrumental aggression; combat sports directly express corporal fighting. The lived experience of combat is oscillatory, dynamically shifting between forms based on affective, empathic, and motivational modulations. Training fosters development by mediating these transitions, cultivating reflection and resilience. Maintaining this structure requires empathic vigilance, affective modulation, and a sensible norm. Understanding this phenomenological specificity grounds the proposition of a Psychology of MA&CS, clarifying how combat promotes ethical development and intersubjective formation by sustaining experiential tension.

## 1 Introduction

Martial arts and combat sports (MA&CS) occupy a paradoxical position in psychological research. Although frequently analyzed through established frameworks—such as stress regulation, motivation, or arousal—these approaches tend to prioritize measurable training outcomes, often neglecting the specificities of fighting as a lived, intentional experience. At the same time, MA&CS are celebrated in popular culture and public discourse for their transformative potential, yet they also evoke controversy, particularly concerning their association with aggression and violence.

Numerous psychological studies have focused on martial arts and combat sports, applying standardized constructs to assess their therapeutic, cognitive, or motivational effects. For example, Willing et al. (2019) examined the use of Brazilian Jiu-Jitsu as a complementary method in the treatment of post-traumatic stress disorder (PTSD) in U.S. veterans, suggesting significant improvements in symptoms related to PTSD, depression, anxiety, and alcohol use. Chen et al. (2019) investigated the mediating role of self-efficacy in the relationship between personality traits and self-control among boxers in China. These examples not only underscore the clinical relevance of combat sports, but also highlight their transcultural significance: an American study on a Brazilian martial art of Asian origin; a Chinese study of a British-origin combat sport. Together, they illustrate the growing recognition of these practices as fields of psychological inquiry across diverse contexts.

Although the field of psychological research on martial arts and combat sports (MA&CS) has grown significantly in recent decades, it still lacks a unified conceptual framework capable of accounting for the specific experiential structures of fighting. This theoretical gap will be addressed through a phenomenological approach in the following sections. Many studies rely on pre-established psychological assessment tools —such as the Self-Efficacy Scale, Resilience Scale, Aggression Scale, Personality Inventories, and Depression Scales—as seen in Xu et al. (2025), Stankovic et al. (2021), Moore et al. (2021, 2023, 2024). Others remain at the level of localized case inquiries, focusing on situated effects or personal beliefs expressed by practitioners, as in Healey et al. (2025) or Jennings and Delamont (2024). While such studies offer valuable insights into the sociocultural and developmental dimensions of these practices, they rarely address the combative experience itself in a rigorous or conceptually integrative manner.

This dispersion of approaches reflects not a failure of individual contributions, but the absence of a shared conceptual framework from which the specific psychological significance of fighting could be clarified. It's no wonder that there is a terminological and practical chaos in martial categories (Martínková and Parry, 2016), which justifies the assertion that, as such, the literature lacks a unified basis for advancing generalizable insights into the lived experience of combat. These conceptual and methodological limitations suggest the need for an approach that does not merely evaluate outcomes, but investigates the structure of the combative experience itself. To address this, the present work proposes an epistemological alternative—grounded in classical phenomenology—that seeks to access the constitutive structures of combative experience as lived and intended by the practitioner. In this line, rather than working from predefined categories or isolated narratives, this approach begins with the experiential field itself. Through phenomenological description, it aims to reconstruct the essential features of fighting practices, its inner tensions, and its developmental possibilities. In doing so, it contributes to a more coherent and experience-based Psychology of MA&CS.

Reviews by Vertonghen and Theeboom (2010) and van der Kooi (2020) illustrate how attempts to evaluate the benefits and harms of these practices tend to oscillate between moralizing judgments and pragmatic assessments of efficacy. While Theeboom found no conclusive evidence regarding psychosocial harm or benefit, van der Kooi identified a predominance of positive outcomes. Yet both studies confirm the persistence of a fundamental question—left largely unexamined: what is the experience of fighting, and how can its structure help us understand the tension between violence, personal development, and ethical formation?

These findings are echoed by a recent body of work by Lafuente et al. (2021, 2024, 2025), who conducted a systematic review and multiple empirical studies on anger in MA&CS practitioners. Their results largely support the trend toward positive psychosocial outcomes, particularly in youth populations and educational contexts. However, like previous reviews, they also emphasize methodological limitations—such as the predominance of cross-sectional designs, reliance on self-reported outcomes, and inconsistent conceptual frameworks—which hinder definitive conclusions. This further reinforces the need for a psychological framework capable of addressing the combative experience itself as a locus of meaning, tension, and potential development.

A systematic review by Ciaccioni et al. (2024) deepens this discussion by examining the relationship between martial arts, combat sports, and a broad range of mental health outcomes. Like previous studies, it highlights the challenges posed by methodological diversity and conceptual inconsistency across the field. Notably, the authors identify key moderators—such as length of training and the pedagogical or competitive context—as central to how practitioners regulate aggression and hostility. The review emphasizes that sustained training fosters self-control, which is essential to the psychological benefits of martial arts. This finding reinforces the need for in-depth experiential approaches such as the one proposed here.

This study builds on a phenomenological perspective grounded in Husserlian philosophy and its psychological developments, particularly in the work of Edith Stein. Rather than treating combat sports as sets of behaviors or therapeutic instruments, this approach aims to clarify how fighting is intended, structured, and experienced. Prior phenomenological studies (Barreira, 2013, 2017a, 2019) have identified a typology of combative experiences—including fighting, brawling, dueling, self-defense, instrumental aggression, and play-fighting—each characterized by distinct intentional meanings and ethical implications.

From this standpoint, the controversies surrounding violence in martial arts—ranging from idealistic rejection (Cynarski, 2022) to utilitarian acceptance (Wetzler, 2015)—miss the essential point: that violence is not allien neither identical to fighting, nor is fighting necessarily violent. Rather, it is the proximity to violence, the risk of its emergence, and the active effort to contain it that give fighting its unique ethical and psychological character. This ambiguity can be further clarified through a phenomenological account of violence, as previously developed (Barreira, 2017b). From this perspective, violence is not merely the infliction of harm, but the rupture of a sensible and embodied norm of encounter. It is a process of affective desubjectivation, whereby the opponent ceases to be perceived as a co-present expressive body and becomes a target of neutralization. Violence, then, is the lived excess that deforms the intercorporeal field of meaning sustained by corporeal fighting. Its emergence signals a breakdown in the bodily reciprocity that gives combat its ethical and dialogical tension. Accordingly, in phenomenology, violence is not defined by the degree of force or physical injury, but by the moment in which the encounter is lived as unacceptable—when the other is no longer perceived as a subject, but as a thing.

As recognized by UNESCO in its designation of traditional martial arts as Intangible Cultural Heritage (Park and Ryu, 2020), the richness of these practices lies not merely in their outward form or therapeutic applications, but in their capacity to engage the practitioner in a disciplined struggle—one that can transform not only bodies, but persons. Through classical phenomenology, this work seeks to illuminate the core structure of that experience, opening the way for a theoretical model that grounds a Psychology of MA&CS in the lived dynamics of fighting itself—a model that is rigorous in its conceptuals scope, yet responsive to cultural specificities, since it focuses on the internal structure of the experience rather than its external forms.

The remainder of this article unfolds in four main sections. First, it outlines the epistemological and methodological foundations of classical phenomenology, emphasizing its relevance for psychological inquiry. Second, it presents a phenomenological typology of combative modalities—such as fighting, brawling, dueling, and play-fighting—based on intentional structures rather than sociocultural classifications. Third, it analyzes the dynamic transitions between these modalities in lived experience, identifying psychological risks and developmental potentials. This phenomenological framework is not developed in abstraction, but in constant dialogue with empirical findings drawn from extensive interviews with practitioners across cultures. Finally, it articulates the implications of this phenomenological approach for building a coherent Psychology of MA&CS, grounded not in prescriptive norms or outcomes, but in the structural complexity of the fighting experience itself.

## 2 Phenomenology as methodology

This study adopts the methodological framework of classical phenomenology, as formulated by Husserl (1970) and developed in its psychological applications by Stein (1989, 2000). Phenomenology does not aim to produce behavioral generalizations or predictive theories, but to clarify the essential structures of experience as they are intentionally lived. At its core, this approach involves a suspension of the “natural attitude”—the uncritical acceptance of objects as pre-given—and the adoption of a reflective stance that allows experience to manifest as it is constituted in consciousness. This shift grants access to a pre-reflective, eidetic layer of meaning, prior to the subject-object dichotomy, where intentional acts and the sense they bestow become accessible to description.

Contrary to approaches that treat intentionality as a matter of subjective perspective, phenomenology reveals intentional structures as intersubjective, eidetic configurations of experience—structures without which the phenomena themselves would not appear as they do. Fighting, in this light, is not a culturally coded action or a psychological behavior to be interpreted, but a form of experience that can be clarified in its constitutive tensions, motivations, and meanings.

To operationalize this approach, the present study draws on empirical-phenomenological research based on interviews with MA&CS practitioners. These interviews are conducted from within a specific interview perspective, grounded in what has been termed suspensive listening. As developed in recent literature (Barreira, 2018; Barreira and Coelho Júnior, 2023), suspensive listening is not merely neutral or open listening, but a deliberate phenomenological posture that suspends interpretive, theoretical, or therapeutic expectations in order to receive what emerges from the first-person experience of the participant.

In practical terms, the epoché and the suspension of the natural attitude are ensured both processually and conclusively. During the empirical phase, the natural attitude is suspended through a systematic identification of habitual concepts, explanations, and generalizations that participants employ to make sense of their experience—these represent the objectifying discourse that overlays the phenomenon itself. In this phase, researchers seek to foster a mode of listening in which the phenomenon is allowed to emerge in its experiential immediacy, that is, as lived and described in first-person perspective, rather than as explained through pre-existing conceptual schemas. At the conclusive moment, the phenomenologist identifies invariant experiential patterns across diverse manifestations through intentional crossing or eidetic variation, thereby evidencing an essential structure. This step is not merely final but reiterative, as the researcher returns to each experiential account to verify whether the proposed structure consistently applies to all of them.

Suspensive listening prolongs and deepens, within a dialogical exchange, the results of the phenomenological analysis of empathy as developed by Edith Stein. It cultivates an attentional stance in which the alterity of the other is not reduced to what can be immediately understood or categorized, but allowed to emerge as a lived and expressive presence. What distinguishes this perspective is its prioritization of first-order speech—the primary articulation of lived experience—over second-order narratives that explain, interpret, or justify it. In other words, the interview becomes a space where the phenomenon speaks for itself, rather than being merely reported or interpreted from the outside. This allows access to the eidetic structure of experience, made possible through the bracketing of the listener's own assumptions and the dialogical openness to the speaker's horizon of meaning.

In this empirical-phenomenological approach, the suspension of the natural attitude is not conceived as a purely mental or theoretical operation, but as a process that unfolds both during the experiential encounter and in the subsequent eidetic analysis. Practically, the *epoché* is exercised first by identifying and bracketing generic notions and explanatory frameworks that obscure the phenomenon's emergence in the lived world. These notions often appear in participants' initial speech and are the correlate of the natural attitude. The interviewer, grounded in an attentive and suspensive listening, progressively redirects the dialogue toward the lived experience itself, encouraging the participant to describe how the phenomenon was experienced, rather than explaining why or what it means. This transition from generic discourse to first-person experiential speech marks the shift from “second-order” to “first-order” expression.

The essential structure, in turn, is not an interpretation of reality but an eidetic result that arises when, through intentional crossing or imaginative variation, one identifies the experiential features that are necessary across multiple manifestations. While any personal experience may include incidental or excessive elements, the essential structure reflects the minimal and constitutive configuration without which the phenomenon would not be what it is. In this sense, phenomenological analysis enables an authentic grasp of direct experience in its prereflective and structural core.

The suspensive listening process of accessing first-order speech—a pivotal step in this approach—relies on a trained attitude of attention in which the researcher actively brackets interpretative reflexes and theories, while remaining empathetically attuned to the speaker's expressive flow. In early segments of interviews, participants often rely on what may be termed “second-order discourse,” where they explain their experience through generalized, conceptual, or impersonal frames. Through suspensive listening, the interviewer recognizes such discourse and gently redirects the inquiry toward concrete, lived episodes—without asking “why” or “for what purpose,” but rather “how” the experience unfolded. When participants shift toward descriptions grounded in their own lived reality, a phenomenological opening occurs: the phenomenon begins to show itself through their embodied language and affective tone, rather than through interpretive rationalizations.

Following this, the method proceeds to a key analytical step known as intentional crossing. This involves comparing the structures of intentionality expressed in different experiential accounts—not to generalize statistically, but to identify invariant elements in how certain experiences, such as fighting or dueling, manifest themselves across different persons and contexts. As described in Barreira (2017a), intentional crossing enables the delineation of typologies grounded not in sociocultural conventions, but in the internal logic of lived experience: the shift from combat to play, the intrusion of hostility into regulated sparring, the withdrawal of ethical engagement from physical interaction.

In this sense, both the interview perspective and the analytical model remain faithful to the transcendental orientation of classical phenomenology. This orientation does not imply a purely reflective or metaphysical posture, but a commitment to investigating experience as it shows itself, including its bodily, affective, and intersubjective dimensions.

Building on this orientation, the present study constructs its typologies through a phenomenological method centered on intentional crossing—a systematic comparison of multiple first-person descriptions of the same phenomenon. This procedure enables the distinction between elements that vary subjectively and those that remain experientially necessary across different manifestations. The resulting eidetic invariants give rise to essential structures and allow for the elaboration of phenomenological typologies that are neither deductive categories nor empirical abstractions, but descriptive syntheses of how a phenomenon—in this case, corporal fighting and its transitions into other combative forms—is constituted in experience. This entire process is grounded in the method of suspensive listening, which grants access to first-order speech and enables the emergence of pre-reflective experiential data.

It is from this territory—what Husserl called the *Lebenswelt* or life-world—that the present work develops its theoretical contribution: a Psychology of MA&CS that is neither reductionist nor moralizing, but grounded in the eidetic intelligibility of fighting itself.

This phenomenological orientation does not exclude psychological or social dimensions of combat, but seeks to clarify how they are constituted within the lived structure of the experience. Rather than starting from predefined categories such as aggression, fear, or gender roles, it returns to the ontological field of encounter, where such dynamics emerge as modulations of embodied and intersubjective engagement. Empirical studies based on this framework have investigated how gender differences and leadership hierarchies appear and interact in the practical experience of fighting (e.g., Figueiredo et al., 2021; Santos and Barreira, in press; Rodrigues et al., 2021; Coelho and Barreira, 2020; Barreira, 2019; Basetti et al., 2016; Melo and Barreira, 2015), revealing how these dynamics are lived, negotiated, and sometimes contested within the intentional structure of combat. The present study offers the eidetic foundation that allows such inquiries to be conducted in a conceptually rigorous and experientially grounded manner.

## 3 The phenomenology of corporal fighting

The phenomenological approach adopted here requires that the experience of combat not be treated as an object of theoretical abstraction or as a simple empirical fact. It is only through the reduction of preconceived definitions and through the return to lived experience that it becomes possible to discern the essential structures underlying combative phenomena. In this context, corporal fighting emerges not merely as a social or physical event but as a field of intentional experiences wherein bodies, wills, and vulnerabilities engage in a singular and immediate confrontation.

Numerous theoretical approaches have sought to define MA&CS based on cultural values, technical systems, historical origins, or social functions. These include evolutionist and naturalist perspectives, such as those from hoplology (comenteded by Bowman, 2017), culturalist or physicalist theories like Tokitsu's (1979, 2000) or Vey's (2010), and sociological-axiological models such as Cynarski's (2019) *General Theory of Martial Arts*, centered on Japanese Budo. In contrast, Bowman (2017), adopting a deconstructionist stance, concludes that martial arts are ultimately indefinable. There is also a terminological eidetic proposal attempting to define martial practices more conceptually (Martínková and Parry, 2016). Yet, in all these cases, the phenomenon of fighting itself—the bodily, lived experience that founds martial practices—remains insufficiently described. It is precisely by returning to this phenomenological foundation that a clarification of combative experiences can be undertaken.

A necessary initial clarification concerns the distinction between combat and beating. While the etymology of combat—“com-battere”, to strike together—already suggests reciprocity, beating (or assault) is characterized by unilateral action, the reduction of the other to passivity or incapacity, and the collapse of any reciprocal horizon. In phenomenological terms, corporal fighting presupposes a minimally shared structure: each participant simultaneously attacks and defends, each recognizing the other as an opposing center of action. Beating, by contrast, dissolves the very conditions that constitute a combative encounter; it is not a degradation of combat but a deviation from its experiential structure.

**Figure 1:**
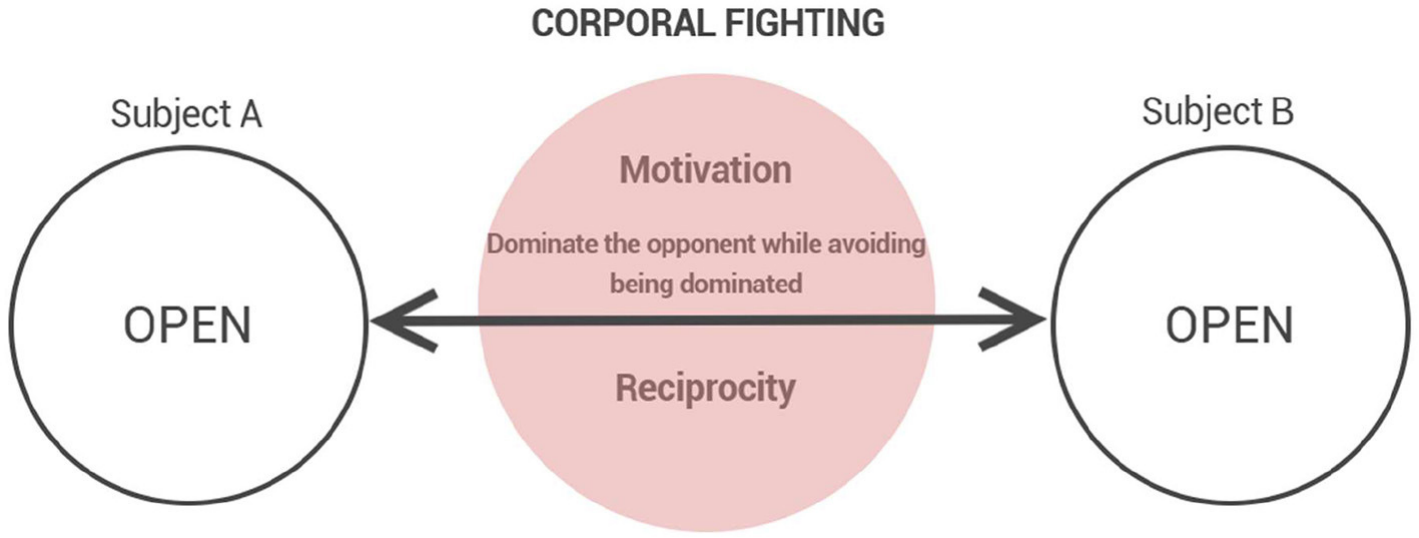
The structure of corporal fighting: reciprocal engagement sustained by motivational tension and empathic openness.

Within this phenomenological field, distinct modalities of combative experience can be eideticly discerned, each presenting a specific configuration of motivational and empathic structures. Corporal fighting, understood in its eidetic purity, is the foundational manifestation of these phenomena. It is defined by the mutual engagement of adversaries, where the intention to dominate and to resist domination coexist as reciprocal and dynamic poles (Figure 1). It is from this basic experiential form that other manifestations such as duels, self-defense, play-fighting, instrumental offensive combat, and brawls can be differentiated.

## 4 Phenomenological clarification of mimetic structures

**Figure 2:**
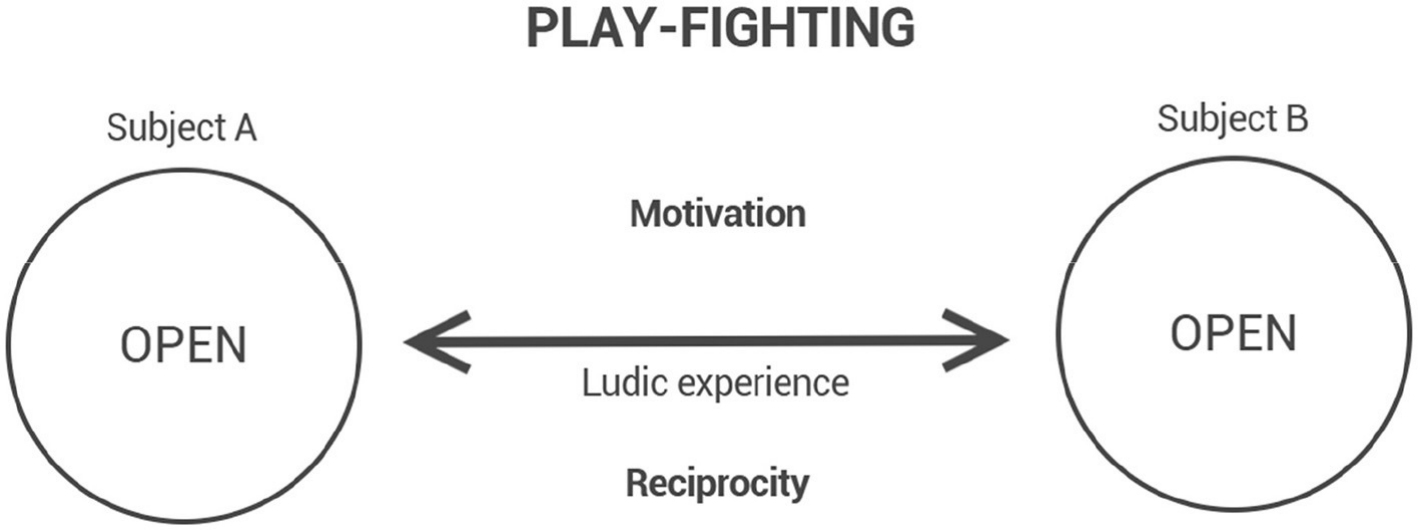
The structure of play-fighting: ludic engagement based on reciprocity and motivational openness.

In the phenomenological approach adopted here, it is crucial to recognize that mimetic gestures, such as those appearing in play-fighting, presuppose a prior layer of presentified experience. Representation does not found combat; rather, combat, in its lived structure, founds the possibility of representation. Thus, before any figurative mimicry, there is a phenomenological realization of fighting, modulated by a ludic intentional horizon (Figure 2). This inversion of precedence—the primacy of manifestation over representation—guides the interpretation of play-fighting not as an imitation of fighting, but as a genuine modality of combative experience.

Although play-fighting may give rise to representations of combat—mimetic enactments in which gestures resemble real fighting—it must not be mistaken for a mere representation. Beneath any figurative layer, there lies a primary expression: a phenomenological realization of combat in a ludic mode. Thus, play-fighting constitutes a form of combat in which bodily engagement remains real, but the intentional horizon is playful. Although gestures and mutual resistance are present, the determination to dominate or to avoid being dominated is relativized under the aegis of play. This indulgence does not eliminate the combative structure; rather, it modulates it, allowing the encounter to unfold within a framework of mutual trust and exploratory engagement. Crucially, this form often occurs asymmetrically: when a more experienced fighter plays at fighting, they allow the other to access the experience of combat, not despite but because of the indulgence of their engagement. It is a central form in pedagogical transmission. On the other hand, in the situations corresponding to the other combative forms described here, play-fighting can sometimes serve as a way of belittling and destabilizing an opponent, thereby emotionally undermining the adversary.

**Figure 3:**
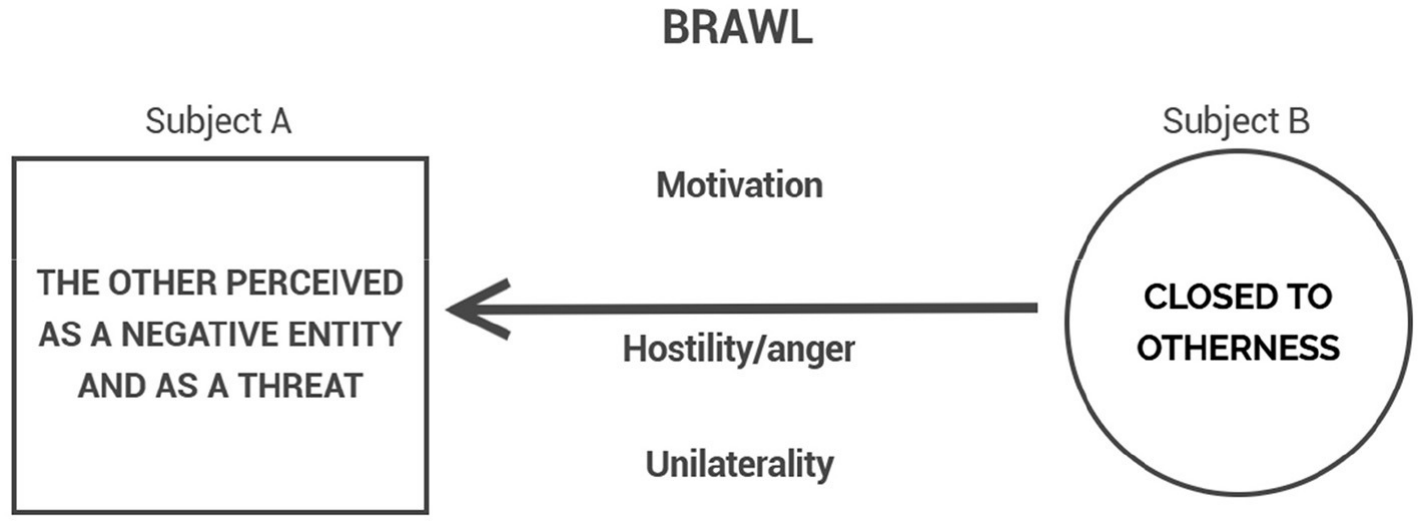
The collapse of combat into brawl: dominance of hostile impulsivity and loss of reciprocal structure.

Brawl manifests a form of combat in which the phenomenological structure of corporal fighting becomes progressively obscured by the predominance of hostile impulsivity, distorting or dissolving the empathic reciprocity that sustains it. The motivational horizon here is dominated by immediate emotional retaliation: the other is no longer recognized as an adversary to be engaged but becomes an object of blind hostility (Figure 3). Sometimes, it is the perception—whether accurate or not—of being the target of blind hostility that triggers an aggressive escalation, leading both parties to brawl. The empathic link that sustains the possibility of combat collapses, totally or partially, depending on the intensity with which hostility obscures the recognition of the other.

**Figure 4:**
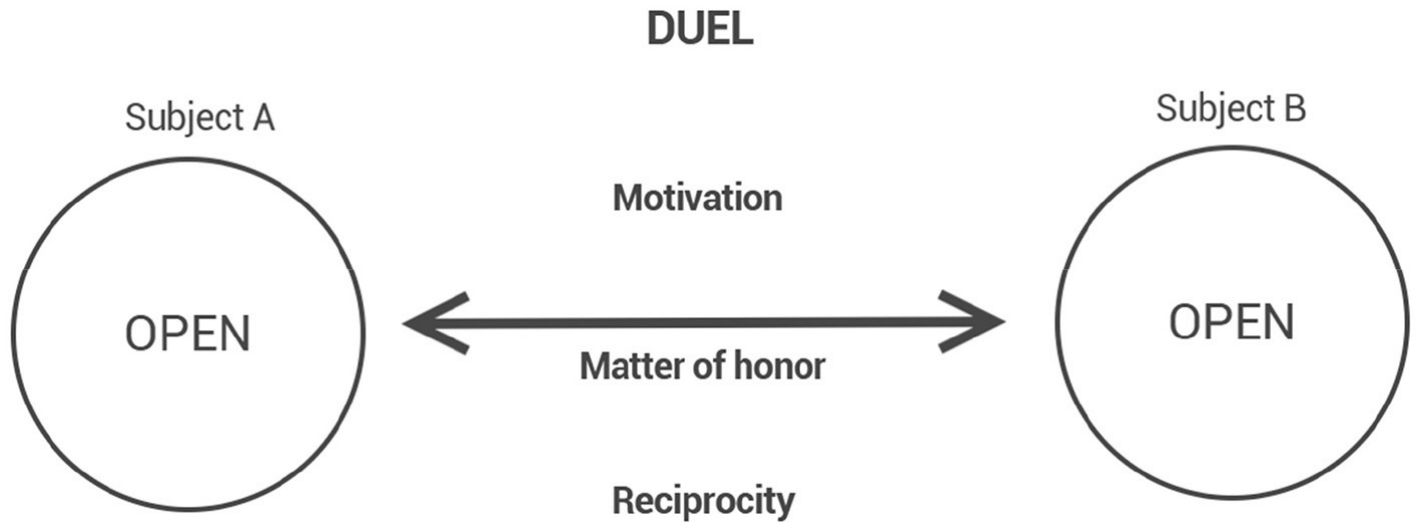
” The Duel, whose experiences structure the founding narrative of martial arts.

Duel, in contrast, is defined by the reframing of hostility through honor. Hostility, though present, is mediated by the institution of a challenge: a formal proposition that re-establishes the adversary as a bearer of honor and moral equivalence (Figure 4). In the context of the duel, honor appears as the symbolic valuation by which bodily existence acquires public meaning. The acceptance of corporeal risk is not an act of mere defiance, but the concrete expression of an individual dignity elevated into the symbolic horizon of reciprocal recognition. Thus, the duel is not simply a combat between bodies, but an affirmation of the honor shared and contested by both adversaries. It is from the experience of the duel that traditional martial arts draw their foundational horizon.

Although different cultural traditions fill the concept of honor with specific values—as in Japanese Bushido or European aristocratic codes—the phenomenological approach adopted here understands honor as a universal intentional structure: a formal self-value that gives existential meaning to the combative experience.

**Figure 5:**
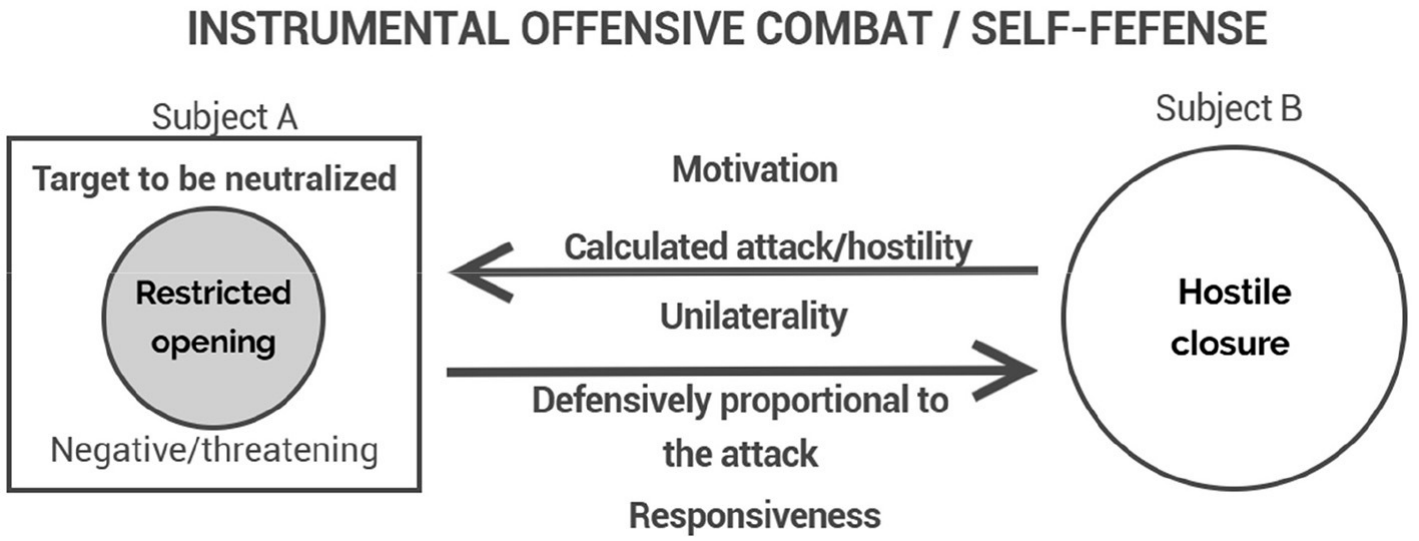
Subject A is in self-defense, requiring responsiveness, which means acting proportionally to the attack suffered. Meanwhile, Subject B attacks unilaterally in the realm of instrumental offensive combat, a calculation that reduces the other to a target to be neutralized, perceived as negative and threatening.

Self-defense emerges where the experience of threat is perceived as illegitimate aggression and calls for a responsive—not merely reactive—violence (Figure 5). The preservation of bodily and patrimonial integrity motivates the combative act, but ethical restraint remains paramount: the use of force must be proportionate and strictly oriented toward neutralizing the threat. The term “modern” here refers not to mere contemporaneity or technological advancement, but to the historical emergence of modernity itself, characterized by the ideal of rule of law and institutional mediation—through police and judiciary—as the legitimate replacements for private violence in conflict resolution. Jigoro Kano's formulation of judo as both a martial art and an educational system exemplifies this transformation, occurring precisely during the shift to the Meiji Era. This was a time when Japan transitioned from a military regime with its distinct castes to embracing a modern state model, although still imperial, influenced by contact with the West. This foundation created a model that would inspire many other contemporary martial disciplines.

Instrumental offensive combat corresponds to a radically different experiential structure (Figure 5). Here, the combative act is driven not by personal conflict or honor, but by strategic or functional necessity: the objective is the elimination or neutralization of a target, pursued according to operational goals that may be institutional, criminal, or individual in nature. Empathy is distorted; the other is reified, no longer apprehended as a subject but treated as an obstacle to be overcome. This modality of combat can manifest in distinct forms: as a criminal act, unscrupulous and antisocial in nature, whether orchestrated by organized groups or carried out by individuals acting with calculated intent; or as a military or police action, operating under a logic of exception to the civil order and typically legitimized by appeals to national security or public safety. In all such cases, the act is marked by its instrumental rationale and by the suppression—or redirection—of ethical reflection. Military martial systems, tactical police training, and certain paramilitary practices are grounded in this instrumental modality.

## 5 Martial systems and foundational forms

The eidetic clarification of combative forms enables the phenomenological foundation of martial systems. Each modality of fighting experience, with its distinct motivational and empathic structures, serves as the experiential root from which different martial practices are historically and culturally constituted.

Even though such structures may appear historically in varied and sometimes overlapping forms, the phenomenological notion of *origin* employed here refers not to historical precedence but to foundational meaning: the duel gives rise to martial systems not because it precedes them chronologically, but because it provides the experiential horizon that renders them meaningful. By contrast, brawling does not ground martial practice but figures as a disruptive limit—what must be contained, avoided, or defended against within martial training. Nonetheless, from a psychological standpoint, many practitioners do take up martial arts precisely after experiencing a brawl or uncontrolled confrontation. The negative resonance of such events may motivate a search for personal development, emotional regulation, and bodily preparedness, both to avoid future escalation and to respond more appropriately, both physically and psychologically, in future encounters. In this way, while brawling is not an eidetic origin, it may serve as an existential trigger for the entry into martial practice.

Traditional martial arts find their origin in the structure of the duel. This analysis suspends the conventional understanding of “traditional” as merely “ancient” or “technically codified,” and instead locates tradition within the sociocultural logic of non-modern societies, where personal honor—inseparable from the honor of one's family or community—demands the readiness to defend it, even at the cost of bodily integrity or life. Within this framework, the figure of the warrior emerges as a paradigmatic expression: one who assumes the decision to fight as an existential and ethical act, in contrast to the soldier, who operates within a depersonalized chain of command. Combat is thus reframed by honor, transforming hostility into a socially recognized contest. Even when ritualized or stylized through technical transmission, traditional martial arts such as *kenjutsu* or ancient *jiu-jitsu* retain the echo of the duel as their founding experience—an experience not necessarily rooted in personal animosity, but in the obligation to uphold a collective moral code through acts of courage and sacrifice.

Modern martial arts are grounded in the experiential structure of self-defense. Violence becomes legitimate only as a last resort, subordinate to the rule of law and proportional restraint. The model established by Jigoro Kano's judo exemplifies this reorganization, where combative techniques are reinterpreted within an educational and civic framework (Kano, 1937).

Military martial systems emerge from the experiential structure of instrumental offensive combat. Strategic functionality overrides interpersonal recognition, and martial action is framed by objectives of neutralization or elimination under imperatives of duty.

Combat sports materialize corporal fighting as a regulated and contractual contest. Stripped of honorific, defensive, or military horizons, sportive combat organizes struggle as a direct confrontation structured by technical rules and competitive challenge.

Although eidetic distinctions are possible, practical martial systems often blend elements from these different horizons. Phenomenological clarification does not seek rigid separation but aims to illuminate the foundational experiential fields sustaining the diversity of martial practices.

Beyond technical, historical, or sociological classifications, phenomenological analysis reveals a deeper structure: all practices that teach, train, and compete in combative modes necessarily organize themselves around corporal fighting. Self-defense, duel, and instrumental offensive combat, while thematically significant, are not in themselves practical modalities but forms of experience that are mimetically evoked within training. Traditional, modern, and military martial arts, as well as combat sports, thus draw on corporal fighting as the concrete, teachable, and trainable expression of combative engagement. It is from this eidetic understanding that the transition to the psychological dynamics of the combat experience becomes necessary: to comprehend the lived reality of practitioners, it is essential to analyze how corporal fighting, along with its possible transitions into brawl or play-fighting, structures their existential engagement within martial practices.

Before proceeding to the analysis of the psychological dynamics inherent to combative experiences, it is necessary to establish a phenomenological typology of the fundamental forms of fighting. Through the application of eidetic reduction, these forms were described by suspending pre-established definitions and returning to the structures of lived experience that constitute them. Table 1 systematizes each form according to its essential motivational and empathic structures, clarifying whether violence is present or absent in each case, and indicating the martial horizons these forms tend to found. This eidetic approach not only reveals the diversity of combative experiences but also demonstrates how the same bodily engagement—corporal fighting—serves as the practical substrate for traditional martial arts, modern martial arts, military martial systems, and combat sports. In practice, these horizons are not mutually exclusive: the same practitioner or the same martial system may, depending on context and purpose, embody overlapping experiential modalities.

**Table 1:** Phenomenological distinctions and martial system structures.

**Form of combat**	**Motivational experience**	**Empathic experience**	**Associated horizon**	**Violence?**
Duel	Defense of honor; confrontation through a challenge	Recognition of the other as bearer of honor; reframing hostility	Traditional martial arts	Yes, consensual and regulated by honor and challenge
Self-defense	Preservation of bodily (patrimonial) integrity in absence of legal mediation	Recognition of other's aggression as illegitimate; defense as justified	Modern martial arts	Yes, responsive and justified for protection of legitimate integrity
Corporal fighting	Desire to dominate without being dominated; mutual engagement	Openness and mutual responsiveness to the other's actions	Formal realization in Combat Sports; Pedagogical base for martial practices involving fighting	No, even if physical degradation is consented through mutual engagement (e.g., MMA)
Play-fighting	Ludic exploration through trust and pedagogical indulgence	Trustful modulation to protect the other's integrity	Training and Learning within Martial arts	No, ludic engagement without intention to harm
Instrumental offensive combat	Fulfillment of duty; elimination or neutralization of threat	Reification of the other; distortion of empathy through functional objectification	Military martial systems	Yes, instrumentalized and directed toward neutralization of the other
Brawl	Hostile impulsivity; immediate emotional retaliation	Gradual or total collapse of recognition toward the other	Disruption of combative structures	Yes, uncontrolled and driven by hostile impulsivity with collapse or partial collapse of recognition

Martial systems are grounded in the lived structure of corporal fighting. In traditional, modern, and military contexts, this structure makes it possible to simulate archetypal combative situations—duel, self-defense, or instrumental offensive combat—by sustaining real engagements whose motivational intensity and ethical posture mirror those foundational forms. These systems do not merely reproduce such forms; they enact them as present lived experiences.

Combat sports, in turn, do not simulate other forms—they express corporal fighting directly, shaping it through normatized structures that preserve its intentional dynamics. While each sport imposes specific rules—karate, for instance, scoring percussive strikes with hands and feet, and judo, throws and immobilizations—these normative frameworks do not alter the combative core but rather configure how it unfolds. Notably, each sport censors the very gestures prioritized in the other, reinforcing its unique internal logic: sport karate prohibits judo's grapples; sport judo forbids karate's strikes.

Although sportive combat does not require mimetic reference to other forms, it may nonetheless evoke their imaginaries. Original point systems in sport karate and sport judo, for example, implicitly echoed the notion of a definitive blow—an ippon or decisive strike—that in duel or self-defense might disable an adversary. In this sense, even normatized sports retain traces of a combat ethos historically rooted in more violent or honor-based forms. It's not uncommon for there to be lament and disapproval when a martial art becomes so sportified that it loses reference to so-called real combat—violent encounters such as duels—prompting institutional returns to traditions that frame the practice as warrior-like rather than merely sportive. Popular culture reinforced this conflation, as in *Karate Kid* (1983), where a personal conflict between teenagers is resolved not through a brawl, but through a sanctioned sportive match that functions as a proper duel, effectively merging the imaginary of personal combat with the ethics of competition.

These contributions may also resonate with recent proposals from the ecological dynamics framework. In particular, the constraints-led approach (CLA) emphasizes the relational coupling of the body, environment, and task, focusing on the emergence of action within dynamic systems (Sánchez-García, 2025; Araújo et al., 2019). Our analysis adds to this approach by integrating a pre-reflective psychological dimension that exceeds the motor domain and accounts for the dynamic modulation of emotional arousal, value orientation, and experiential engagement in combat. This psychological dynamic, with its oscillations toward either aggression or playful resonance, allows for a refined understanding of the fight experience and offers a phenomenologically grounded articulation with the ethical narratives embedded in different martial cultures.

This eidetic clarification sets the stage for understanding how these combative structures unfold psychologically in real-life practice—a task addressed in the following section.

## 6 Psychological dynamics of the combat experience

### 6.1 Introduction: the oscillatory nature of combat experience

In the phenomenological perspective adopted here, corporal fighting is never a static or neutral experience. It constitutes an existential tension, where bodies and wills intertwine in a continuous process of mutual testing and self-exposure (Figure 1). Beneath the corporeal and volitional acts—motor control and consciously taken decisions—lie psychic acts that pre-reflexively determine the field of behavior, sustaining the experiential basis upon which combat unfolds. These oscillations do not merely accompany combat; they are intrinsic to its phenomenological reality—making pedagogical mediation and psychological resilience central to martial learning.

Within this layered structure, the lived experience of combat is not fixed: it oscillates according to motivational intensities, emotional fluctuations, and empathic modulations. Rather than a monolithic act, corporal fighting constitutes a dynamic field of experiential possibilities. At any given moment, the horizon of the encounter may tilt toward an escalation of hostility, culminating in the collapse of empathic recognition and the emergence of brawl (Figure 3). Conversely, the intentional intensity that sustains combat may slacken, allowing a ludic modulation to take hold, giving rise to play-fighting (Figure 2). These oscillations do not merely accompany combat; they are intrinsic to its phenomenological reality.

**Figure 6:**
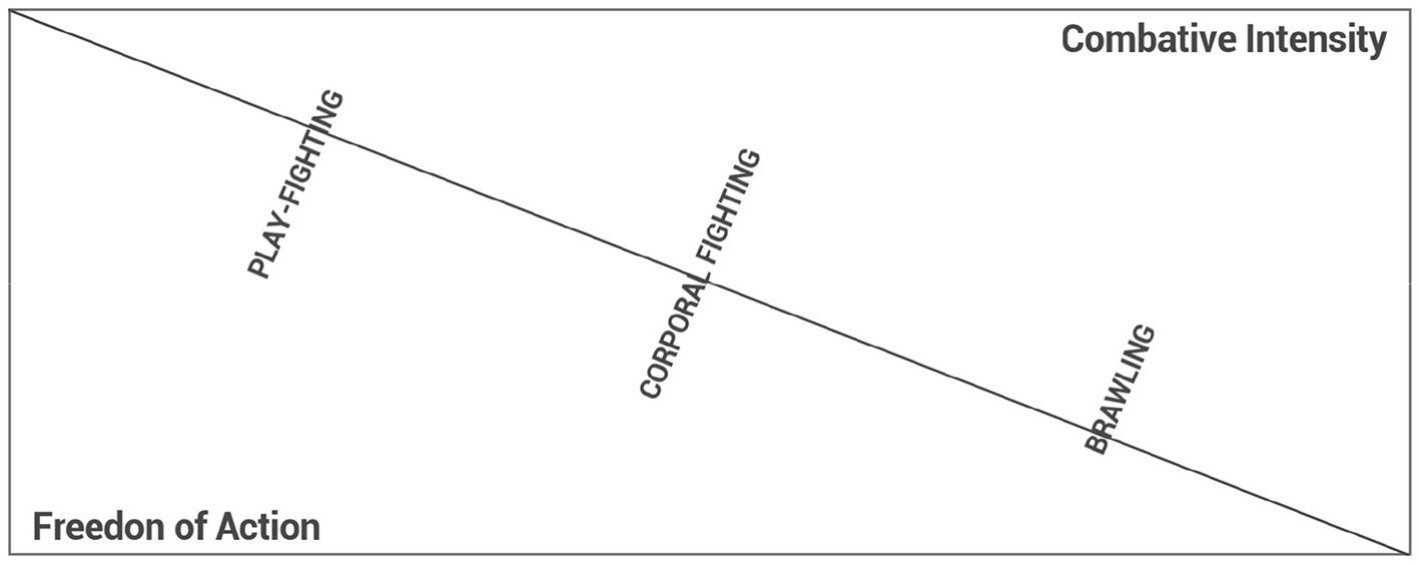
” The dynamics of corporal fighting. As combative intensity increases, freedom of action decreases, leading to a greater likelihood of brawling. Conversely, reduced combative intensity with greater freedom of action corresponds to a tendency toward play-fighting. Corporal fighting is always at risk of destabilizing into these combative forms. Diagram adapted from Miranda and Barreira (2022).

Figure 6 illustrates the oscillatory structure of psychological dynamics in combat, tracing transitions between play-fighting, full engagement, and brawl, as shaped by affective, empathic, and normative modulations.

Fighting is not merely a matter of executing techniques or seeking dominance; it involves sustaining, under affective, cognitive, and ethical pressures, a specific intentional structure—one that recognizes the other as a combatant and oneself as co-engaged in a shared encounter. This maintenance is fragile: it requires resilience against both the seductive pull of hostile impulsivity and the centrifugal tendency toward ludic dispersion. The phenomenological dynamics of combat thus unfold between poles of intensification and distension, with the fighter's existential position continuously negotiated between the gravity of confrontation and the levity of play.

Understanding this oscillatory nature is essential for grasping the psychological challenges inherent in martial practices and combat sports. It is not merely the presence of physical risk that shapes the experience but the constant risk of existential drift: the possibility of falling into violence, or of losing combativeness in favor of play. It is in this subtle terrain that the art of fighting is forged, not only as a technical skill but as an ethical and affective practice.

### 6.2 Foundational experiential dynamics

The experience of corporal fighting is sustained by a foundational dynamic tension. At every moment of the encounter, the fighter is immersed in a structure that requires the maintenance of reciprocal engagement: not merely acting or reacting, but continuously acknowledging the other as an active and responsive subject within the shared field of confrontation.

This intentional structure is fragile and dynamic. Beneath the visible gestures and the consciously directed will, pre-reflexive psychic acts operate, shaping the field of combativity even before deliberate reflection takes place. These acts manifest as affective, motivational, and empathic currents that modulate the intensity of engagement, the openness to the other, and the interpretation of unfolding events.

Corporal fighting, phenomenologically understood, is not reducible to technical exchange or competitive drive. It is an intersubjective phenomenon where each fighter's experience is co-constituted by the presence, resistance, and expressivity of the other. To fight is to enter a reciprocal structure of challenge and response, testing not only bodily abilities but the capacity to sustain a lived tension where the other is simultaneously an adversary and a co-constitutor of meaning.

The phenomenological analysis reveals that this tension can fluctuate even within a single bout. Minor failures in motor control, emotional regulation, or empathic attunement may destabilize the intentional structure of combat. This destabilization predisposes the experience to drift in two opposing directions: escalating into brawl, or softening into play-fighting. In both cases, the phenomenological reality of corporal fighting is threatened—not necessarily by external factors, but by shifts internal to the lived experience of the fighters themselves.

Thus, understanding the foundational experiential dynamics of combat requires not only an analysis of actions and techniques, but a description of the lived oscillations that precede and determine them. It is through the modulation of affective, motivational, and empathic fields that the practice of fighting emerges either as a disciplined and enriching experience, or as an occasion for violence or dispersion.

Recent findings from a comprehensive, ongoing research project conducted with practitioners from eight different MA&CS in Brazil confirm the fragility of this structure. Drawing on extensive interviews with athletes, this work highlights that the stability of combat experience is constantly challenged by shifts in emotional intensity, attentional focus, and empathic engagement. This growing body of research—now extending to France, Portugal, and Spain—has revealed that even advanced practitioners may experience breakdowns in the lived structure of fighting, particularly in what are now called “disruptive situations” (Santos and Barreira, in press).

### 6.3 Phenomenological transitions

The lived experience of corporal fighting is structured by a precarious equilibrium. These “disruptive situations” emerge in experience as thresholds where the intentional structure of corporal fighting undergoes sudden destabilization, sometimes escalating to psychological or physical rupture. In the experience of MA&CS practitioners, such moments are not merely technical failures but existential thresholds that may reveal ethical, emotional, and pedagogical dimensions of combat previously unacknowledged (Santos and Barreira, in press). Such observations resonate with sociological studies on consent dynamics in combat sports, particularly those emphasizing how third parties—such as coaches, referees, and spectators—play a crucial role in the intersubjective regulation of combat encounters (Channon and Matthews, 2021). At every moment, the intentional engagement with the opponent can either intensify toward hostile impulsivity or soften into a ludic modulation. These transitions, while phenomenologically possible at any point during the encounter, do not occur arbitrarily: they respond to shifts in affective regulation, empathic attunement, motivational tension, and the capacity to sustain the lived structure of combat.

When the tension inherent in corporal fighting slackens, and the determination to dominate or resist domination ceases to structure the encounter, the experience tends to shift toward play-fighting. This ludic modulation is not simply a relaxation of motor intensity; it represents a phenomenological distension in which the acts of attacking and defending are no longer governed by the serious intentionality of combat, but by an indulgent openness to experimentation, exploration, and shared enjoyment. In play-fighting, the opponent is still recognized as such, but the stakes of engagement are diminished: gestures lose their imperative weight, allowing for a playful, sometimes asymmetric dynamic in which mutual responsiveness remains, but without the demand for full existential commitment. This structure was particularly observed among Brazilian Jiu-Jitsu practitioners, for whom playfulness emerges especially when skill gaps allow one practitioner to “open space” for the other to explore techniques in ways that would be unlikely under combative pressure (Basetti et al., 2016).

This shift may occur deliberately, as in pedagogical situations where an experienced fighter modulates the engagement to facilitate the learning of a novice, or involuntarily, as a psychological response to the difficulty of sustaining combative tension under pressure.

Conversely, when affective regulation fails in the opposite direction, and hostile emotions such as frustration, humiliation, or fear are allowed to dominate the field of experience, the intentional structure of corporal fighting risks collapsing into brawl. In this case, the opponent ceases to be recognized as a co-constituting subject within the combative encounter and becomes perceived primarily as an obstacle or enemy to be neutralized. In Brazilian Jiu-Jitsu, this transition is marked by a shift in perception where the opponent ceases to be experienced as a legitimate co-combatant and becomes emotionally framed as a threat, provoking disproportionate or careless reactions even when no deliberate intent to injure exists (Basetti et al., 2016).

The collapse of empathic recognition leads to a distortion of intersubjectivity: the other is no longer a partner in the combative relation but is reduced to an object of attack. Importantly, this collapse can occur even if the external forms of combat—strikes, defenses, counterattacks—are still performed; phenomenologically, what changes is the underlying intentionality that sustains the experience.

Such transitions have been observed and analyzed across empirical phenomenological studies in Capoeira (Melo and Barreira, 2015), Brazilian Jiu-Jitsu (Basetti et al., 2016), Greco-Roman wrestling (Coelho and Barreira, 2020), and professional MMA (Rodrigues et al., 2021), highlighting the contingent and oscillatory nature of the fighting experience. *The transitions in question are not all-or-nothing phenomena*. Between the poles of full corporal fighting and brawl, or between corporal fighting and play-fighting, there exist intermediate zones where the intentional structure fluctuates, momentarily stabilizing or threatening to tip over. Fighters may oscillate between moments of playful indulgence and renewed combative focus, or between controlled aggression and surges of hostile impulsivity. Recognizing and describing these micro-fluctuations is crucial for understanding the real psychological demands of martial practice: sustaining the intentional structure of corporal fighting is not a given but an achievement, constantly tested by the oscillations inherent to the lived experience of combat.

The detailed analysis of these transitions reveals that martial training must address not only technical and tactical proficiency but also the cultivation of affective resilience and empathic regulation. Learning to fight, in this sense, means learning to dwell within the tension of combat without collapsing into violence or dissipating into play, maintaining the existential posture that defines the art of fighting itself.

## 7 Protective tendencies and experiential stabilizations

The maintenance of the intentional structure of corporal fighting, amid oscillations of tension and affective intensity, is not spontaneous. It is the result of experiential dynamics that allow the fighter to sustain the shared field of combat, avoiding both collapse into violence and dispersion into play. Phenomenologically, what is at stake are not “factors” in the empirical sense, but lived tendencies, motivational orientations, and modes of empathic attunement that either preserve or undermine the specific experience of combat.

One central experiential stabilization is empathic vigilance—the lived disposition to recognize the opponent as a co-present subject within the combative structure. As studies in capoeira and wrestling practices have shown (Melo and Barreira, 2015; Coelho and Barreira, 2020), sustaining empathy under the pressures of confrontation is crucial for maintaining the field of combativity without falling into hostility or disconnection. This empathy is not merely reflective; it is pre-reflexively anchored in the bodily experience of the other's movements, reactions, and vulnerabilities.

Closely related to empathic attunement is affective modulation: the dynamic regulation of emotions such as fear, anger, pride, or humiliation within the experience of combat (Barreira, 2017a,b, 2019). Fighters who succeed in maintaining the intentional structure of corporal fighting are those who do not suppress emotions but integrate them into the ongoing experience, preserving the adversary as an interlocutor rather than reducing him or her to an object of attack or avoidance. This process has been described in interviews with capoeira and MMA practitioners, who report learning to “listen” to the intensity of the other's presence, adapting themselves to avoid disconnection or escalation (Barreira, 2019; Rodrigues et al., 2021).

The progressive acquisition of experience also shapes the phenomenological field. Through repeated exposure to combative situations, practitioners develop a refined sensitivity to the thresholds of escalation and distension (Rodrigues et al., 2021; Telles et al., 2018). These modulations were observed in Jiu-Jitsu, where experienced fighters described a practical awareness of “boundaries” between combative forms as essential to preventing experiential drift (Basetti et al., 2016). To some degree, in all the MA&CS surveyed, experienced practitioners learn to perceive early shifts in motivation, empathic openness, and affective charge. This enables them to adjust their engagement and maintain the shared structure of fighting, rather than letting it dissolve into brawl or play-fighting.

These findings resonate with the hypothesis proposed by Ciaccioni et al. (2024), which suggests that the potential of MA&CS to reduce hostility and foster emotional regulation may depend on the continuity and meaningfulness of training. Our analysis deepens this claim by showing that such psychological effects are not merely behavioral outcomes, but are rooted in the lived intentional structure of corporal fighting itself. When this structure is stabilized through pedagogical orientation and normative guidance, it cultivates embodied dispositions of self-regulation, empathic vigilance, and ethical engagement (Barreira, 2017a).

Finally, the presence of a lived normative structure—what phenomenological analysis terms a “sensible norm” (Barreira, 2017b)—anchors the experience of fighting as an ethical and non-violent practice, even amid intense physical confrontation. This “sensible norm” operates pre-reflexively, structuring the encounter as a field of mutual recognition and consent, rather than domination or annihilation. This dynamic was exemplified in interviews with professional MMA fighters, who, despite the extreme intensity of the sport, described their matches not as violent clashes but as intersubjective events of mutual commitment, responsibility, and respect—encounters structured by ethical awareness rather than antagonism (Rodrigues et al., 2021; Barreira, 2019). Understanding how this delicate equilibrium is lived, threatened, and sometimes restored during practice forms the core of the psychological dynamics we now explore.

Originally developed to address the ambiguity between combative excellence and violent collapse in sports practice, the notion of a “sensible norm” refers to a pre-reflective, intercorporeal horizon of affective and ethical attunement. It is not reducible to codified rules or external conventions, but arises from the embodied interaction itself—shaping what is lived as acceptable or excessive, respectful or annihilating, within a given combative engagement (Barreira, 2017b). This norm is both individual and shared: it emerges from the mutual recognition of vulnerability, resistance, and expressive gesture, and is constantly at risk of rupture when the opponent ceases to be seen as a person and becomes merely a target or obstacle. Crucially, this rupture may occur not only in action but also in perception: a fighter may feel dehumanized or objectified—even if this was not the other's intention—leading to a breakdown in the lived reciprocity that sustains the combative encounter.

These insights align with Ciaccioni et al.'s (2025) opinion, who emphasize that the pedagogical and philosophical values embedded in martial arts—such as emotional control, mutual respect, and ethical guidance—form a unique framework for promoting psychological wellbeing. Our analysis specifies how such benefits are grounded in the experiential structure of fighting itself, especially when training is sustained within ethically oriented environments.

Significantly, interviews reveal that the aftermath of disruptive situations often becomes a privileged moment for personal development, when practitioners, supported by their instructors, reinterpret the meaning of their combative experience. This points to a pedagogical role of rupture in martial arts: not as an endpoint, but as a transitional threshold that, when adequately mediated, fosters psychological growth, ethical reflection, and deepened commitment to the practice (Santos and Barreira, in press).

### 7.1 Phenomenological and psychological implications for martial training and development

The phenomenological analysis of the lived structures of corporal fighting reveals that engaging in combative practices is not merely a matter of external technique, motor skill, or tactical proficiency. Rather, it involves sustaining a delicate intentional posture, a pre-reflective horizon in which the opponent is simultaneously resisted and recognized, challenged and respected. These findings resonate with recent interdisciplinary perspectives that underscore the transformative potential of combat sports to foster individual and community wellbeing across physical, psychological, and social domains (Ciaccioni et al., 2025). Our phenomenological approach complements this view by clarifying how such developmental effects are anchored not merely in behavioral outcomes, but in the intentional structure of corporal fighting—where reciprocity, challenge, and ethical recognition are dynamically sustained.

The specificity of corporal fighting lies precisely in this lived tension: the fighter must maintain the dynamic orientation of struggle without collapsing into hostile violence (brawl) or dissolving into playful disengagement (play-fighting). Every combative encounter thus constitutes a test of existential balance, where affective regulation, empathic attunement, and motivational integrity must be maintained against the constant risk of experiential collapse.

From a psychological standpoint, this phenomenological insight implies that the development of martial competence cannot be reduced to external behaviors or mental attitudes detached from the lived body. Training in combat sports and martial arts necessarily involves the formation of embodied intentionalities: lived dispositions to recognize, regulate, and modulate affective impulses within the flux of interpersonal engagement.

Furthermore, the preservation of the combative structure requires the internalization of a sensible norm—a pre-reflective, intersubjectively sustained standard that orients behavior toward reciprocity, consent, and mutual respect even under conditions of physical confrontation. The capacity to inhabit this norm sensitively, rather than merely adhering to external rules, constitutes the ethical core of martial practice.

Phenomenologically, fighting is not inherently violent; it becomes violent when the intentional structure that sustains the reciprocity of combat breaks down. Understanding this dynamic allows for a refined psychological appreciation of the processes by which martial training fosters not only physical prowess but also emotional resilience, ethical awareness, and intersubjective sensitivity.

These findings reinforce the need to understand martial arts as not merely physical disciplines, but as complex intersubjective practices where failure, rupture, and emotional intensity are integrated into a broader pedagogical horizon. The ability to reintegrate oneself after a disruptive episode becomes, in this sense, a measure of psychological resilience and existential maturity (Santos and Barreira, in press).

These findings resonate with qualitative research by Healey et al. (2025), which explored how Australian MA&CS practitioners perceive the impact of their practice on personal wellbeing. Their study highlights how experiences of discipline, embodied engagement, and interpersonal challenge in combative settings “cross over into the rest of life,” fostering self-awareness, emotional resilience, and social connection. While the study emphasizes narrative and relational dimensions, our phenomenological approach clarifies how such outcomes are not simply consequences of context or culture, but emerge from the lived structure of corporal fighting itself. By elucidating the intentional dynamics of fighting—where challenge, reciprocity, and ethical orientation are sustained—our model provides a systematic and theoretically grounded framework to explain how such localized outcomes may arise.

Thus, MA&CS, when approached from the perspective of lived experience, reveal themselves as privileged fields for the cultivation of human development. They embody a practical education of the body, the emotions, and the relational self, demanding from the practitioner not only technical mastery but existential maturity—the ability to fight without hatred, to resist without dehumanizing, and to affirm oneself without annihilating the other.

## 8 Conclusion

This theoretical framework is not an endpoint, but the foundation of an ongoing international research program, organized as a network involving the authors and their respective institutions, with plans to extend the program to other continents, such as Africa. Grounded in the phenomenological model developed here, this program seeks to identify the psychosocial processes that mediate how combat practice contributes to personal development, ethical formation, and practitioner continuity. Coordinated across a research network involving institutions and scholars from Brazil, Portugal, France, and Spain—with hundreds of interviews already conducted and further collaborations expanding to other countries—this effort aims to clarify how affective transitions, normative ruptures, and interpersonal recognition are shaped by different martial cultures, pedagogical interventions, and contextual factors. Special attention is devoted to the trajectories of women practitioners, whose experiences often involve negotiating complex dynamics of recognition, empowerment, and belonging—challenges that have been documented in recent studies exploring exemplary and harmful practices in martial instruction (Figueiredo et al., 2021). The results of this program are expected to offer transcultural insights directly connected to the lived experience of practitioners and instructors, thereby supporting the development of practices and guidelines with immediate pedagogical applicability. The ultimate goal is to refine a model of psychosocial development anchored in the lived experience of fighting, capable of identifying the experiential conditions that foster or hinder its transformative potential.

Building on the preceding analyses, this article has proposed a phenomenological understanding of MA&CS as practices grounded in the lived structure of corporal fighting. Through eidetic analysis and empirical investigation, we have shown that the specificity of combative experience does not reside in violence, but in the intentional configuration that sustains a shared and reciprocal confrontation. The concept of corporal fighting—distinct from brawl, self-defense, or instrumental violence—offers a foundation from which martial systems, in their various cultural and institutional forms, emerge and evolve.

By distinguishing five essential forms of combat and showing how traditional, modern, and military martial systems are structured through the mimetic enactment of duel, self-defense, and instrumental offensive combat, respectively, the analysis locates corporal fighting as the practical and phenomenological core that allows these systems to train, simulate, and refine combative experiences. In contrast, combat sports do not necessarily mimic other forms: they express corporal fighting itself, shaping it through normatized rules that preserve its intentional structure, even when their scoring systems implicitly evoke archetypal combats such as duels or acts of self-defense.

The empirical studies reviewed demonstrate how the intentional structure of combat can oscillate during practice, producing transitions that challenge the existential equilibrium of the fighter. These modulations—whether subtle or disruptive—are not marginal to the experience; they define the psychological challenges and developmental potential of martial practice. When adequately mediated by instructors, these oscillations, especially those triggered by rupture, become formative moments: opportunities for ethical reflection, emotional maturation, and renewed commitment to the practice.

It is precisely the phenomenological specificity of corporal fighting—its reciprocal, tension-sustained intentional structure—that makes it vulnerable to collapse into violence or dispersion, but also capable of fostering ethical development. When this structure is preserved and supported pedagogically, the fighter learns to dwell within conflict without succumbing to hostility or volatility. In this sense, it is not by eliminating risk but by inhabiting it ethically that corporal fighting becomes a medium for psychological formation.

In contrast to theoretical models that reduce martial arts to cultural codes, sociological constructs, or behavioral routines, the phenomenological perspective developed here affirms the primacy of lived experience. Even in comparison with recent sociological analyses of consent in combat (Channon and Matthews, 2021), the phenomenological method adds a necessary depth by clarifying how the intersubjective structure of fighting is sustained from within. The concept of a “sensible norm” articulates this from the inside out, as a pre-reflective and ethically charged horizon of perception and action.

What emerges, therefore, is not merely a theory of combat or a psychological account of martial arts, but the foundation of a field: a Psychology of MA&CS. Grounded in phenomenological analysis and enriched by empirical investigation, this field articulates the experiential specificities of corporal fighting across diverse cultural and institutional modalities. It offers a systematic framework for understanding their psychological and developmental dynamics, becoming a path not of domination, but of ethical growth and intersubjective formation.

Finally, this proposal contributes a much-needed phenomenological account of how combative practice becomes psychologically formative—not by requiring rupture, but by sustaining experiential tension as a field of ethical and emotional development. In doing so, it offers an original response to key demands in the literature for conceptual clarity on the psychological and pedagogical mechanisms underlying martial arts' impact on wellbeing. Rather than reducing combat sports to behavioral outcomes or social categories, our approach foregrounds the lived structure of fighting as the experiential core from which transformative effects may emerge.

## Data Availability

The original contributions presented in the study are included in the article/supplementary material, further inquiries can be directed to the corresponding author.
